# Myocarditis Following Invasive Group A *Streptococcus* Infection: Important Differential Diagnostic Distinctions

**DOI:** 10.1155/crdi/5349221

**Published:** 2026-04-06

**Authors:** Jonathan M. Oxman, Michael Bourne, David C. Perlman, Nicholas Sells

**Affiliations:** ^1^ Department of Medicine, Mount Sinai Morningside-West Hospital Center, Icahn School of Medicine at Mount Sinai, 440 West 114, ^th^, Street, New York, 10025, New York State, USA, mountsinai.org

**Keywords:** acute rheumatic fever, Group A *Streptococcus*, nonrheumatic streptococcal pharyngitis−associated myocarditis, SPAM, *Streptococcus pyogenes*

## Abstract

Nonrheumatic streptococcal pharyngitis−associated myocarditis presents as acute myocarditis within days of Group A *Streptococcus* pharyngitis, typically resolving with antibiotics and minimal sequelae. It is thought to result from direct bacterial toxin injury rather than autoimmunity. Little epidemiologic data regarding its incidence and prevalence exist, and standardized diagnostic criteria are lacking. In contrast, acute rheumatic fever develops 2−4 weeks after a Group A *Streptococcus* pharyngitis and is autoimmune‐mediated. Diagnosis is based on the revised Jones criteria, and only carditis leads to sequelae, including rheumatic heart disease. We report the case of a 21‐year‐old male, in a high‐resource setting, with invasive Group A *Streptococcus* who developed carditis with mitral regurgitation and heart failure, for which nonrheumatic streptococcal pharyngitis−associated myocarditis and acute rheumatic fever with carditis were considered. This case highlights the need for clinical differentiation between, and further study of, both entities.

## 1. Introduction

Nonrheumatic streptococcal pharyngitis−associated myocarditis (SPAM) is an incompletely defined condition characterized by acute onset myocarditis within days of Group A *Streptococcus* (GAS) pharyngitis, possibly mediated by direct bacterial toxin−induced injury rather than autoimmunity [1]. SPAM often resolves with antibiotics and minimal sequelae but may be underrecognized due to a lack of epidemiologic data and standardized diagnostic criteria, leading to misdiagnosis and underdiagnosis [1–4].

Acute rheumatic fever (ARF) is an autoimmune complication of GAS pharyngitis, often presenting 2−4 weeks after infection and diagnosed based on the revised Jones criteria [5–7]. Arthritis and carditis are the two most common presentations of ARF, but only carditis leads to chronic sequelae including rheumatic heart disease (RHD) [5, 8, 9]. Early antibiotic treatment of GAS pharyngitis can decrease the risk of ARF by up to 80%, while penicillin prophylaxis likely reduces the risk of recurrent ARF and RHD [9, 10].

Here, we report the case of a 21‐year‐old male presenting, in a high‐resource setting, with a GAS infection who subsequently developed carditis with moderate to severe MR and concomitant heart failure, for which SPAM and ARF with carditis were considered.

## 2. Case Presentation

A 21‐year‐old male who had been hospitalized and discharged three weeks previously for GAS pneumonia and bacteremia presented to the emergency department (ED) with three days of new‐onset worsening dyspnea, orthopnea, chest pain, cough, and facial and bilateral lower extremity swelling.

Prior to his initial admission for GAS pneumonia and bacteremia, he had presented to the ED 4 days prior with right upper quadrant pain worse with inspiration and diffuse body aches; there was no chest pain or dyspnea. He had tachycardia at 120 s beats/minute, fever of 102 degrees Fahrenheit, 98% oxygen saturation on room air, and a blood pressure of 123/73 mmHg. Physical examination was remarkable only for mild right upper quadrant and epigastric tenderness without rebound or guarding. White blood cell count (WBC) was 14.4 K/μL with 80.6% polymorphonuclear leukocytes (PMN). Comprehensive metabolic panel (CMP) was within normal limits. Creatine phosphokinase (CPK) was 33 U/L (within normal limits). Neither a troponin nor an EKG was collected. Nasal polymerase chain reaction (PCR) for respiratory syncytial virus (RSV), SARS‐CoV‐2, and Influenza A and B were negative. Computed tomography (CT) of the abdomen and pelvis was unremarkable. The lung bases were clear, and no pleural or pericardial effusion was present. After fluid resuscitation, acetaminophen, and ketorolac, his fever and tachycardia resolved, and his symptoms improved. In the ED, he did not receive any antibiotics and was discharged the following day with a suspected viral syndrome.

Four days following his initial ED visit, he presented a second time with fever, chills, cough, and pleuritic chest pain, without associated dyspnea, diarrhea, dysuria, or upper respiratory symptoms. During the second encounter, he had a fever of 102.7 degrees Fahrenheit and tachycardia of 110 beats/minute with a normal oxygen saturation. He was ill‐appearing, diaphoretic, and tender in the epigastric and right upper quadrant regions, with diminished breath sounds in the posterior base of the right lung. His exam was otherwise unremarkable, without pharyngitis, exudates, heart murmurs, crackles, joint swelling, edema, or skin rash. The initial WBC was 11.2 K/μL with 86.7% PMNs. The CMP, venous blood gas, and lactate were unremarkable. Urinalysis showed six red blood cells (RBCs)/high power field (HPF), three WBCs/HPF, and was negative for leukocyte esterase and nitrites. Erythrocyte sedimentation rate (ESR) and C‐reactive protein (CRP) were elevated to 94 mm/hr and 276.8 mg/L, respectively. Blood cultures were obtained, and a CT of the abdomen and pelvis demonstrated a right lower lobe pneumonia and a small right pleural effusion, but no intraabdominal pathology. A chest X‐ray (CXR) showed a consolidation in the right lower lobe of the lung and a small parapneumonic effusion consistent with pneumonia. No EKG was obtained. Nasal PCR for RSV, SARS‐CoV‐2, and Influenza A and B were negative, as was his Legionella urine antigen. A 4^th^ generation human immunodeficiency virus (HIV) test was nonreactive. He was started on azithromycin and piperacillin−tazobactam.

Gram stain of the blood cultures showed gram‐positive cocci in pairs and chains after 12 h in two sets. The following day, GAS was identified in blood cultures. A rapid GAS PCR of the pharynx was positive. Throat culture did not show any organisms on gram stain, and no pathogens grew. The following day, he defervesced, and his leukocytosis resolved. Piperacillin−tazobactam was discontinued, and ceftriaxone and clindamycin were initiated. The GAS isolated from blood cultures was susceptible to ceftriaxone and penicillin with minimum inhibitory concentrations (MICs) of ≤ 0.016 and ≤ 0.002, respectively. Repeat blood cultures were negative. On Day 5, the patient was discharged home with oral linezolid 600 mg twice daily to complete a 14‐day course from the first date of negative blood cultures.

Three weeks after being discharged (bringing us to the current admission), he presented for a third time with 3 days of progressive dyspnea, orthopnea, chest pain, cough, and facial and bilateral lower extremity swelling. He had inconsistently taken the linezolid, believing it caused his legs to swell. He had no fever, chills, headache, vision changes, sore throat, upper respiratory symptoms, abdominal pain, nausea, vomiting, diarrhea, or dysuria. In the ED, he was afebrile, with systolic and diastolic blood pressures in the 140 s and low 100 s mmHg, respectively. His heart rate ranged from 70 to 90 beats/minute, and oxygen saturation ranged between 94% and 100% on room air. His initial exam was only notable for decreased breath sounds in the base of the right lung. No heart murmurs were present. His hemoglobin was 13 g/dL, and his WBC was within normal limits. His basic metabolic panel and hepatic function panel were within normal limits. ESR was 29 mm/hr and CRP 3.6 mg/L. Urinalysis showed 108 WBCs, moderate leukocyte esterase, > 182 RBCs, 50 mg/dL of protein, and a total protein to creatinine ratio of 217 mgTP/gCrea. A single high‐sensitivity Cardiac troponin I was 4 ng/L. Respiratory pathogen panel, SARS‐CoV‐2, and influenza PCRs were all negative, as was a Legionella urine antigen.

The patient’s family was originally from the Dominican Republic. He grew up and lived in New York City with his family. He worked as a mail delivery driver, did not travel or hike recently, had a pet turtle, was sexually active with three female partners over the past year, and denied any illegal substance use. He had no recent sick contacts and no known past medical history.

Serial EKGs showed PR intervals between 118 and 152 ms (within normal limits) and nonspecific ST segment changes. CXR demonstrated worsening right lower lobe pneumonia and increased size of the parapneumonic effusion seen on prior admission. Chest CT demonstrated a moderate right‐sided pleural effusion, a small left pleural effusion, and bilateral ground‐glass opacities. Intravenous vancomycin, ceftriaxone, and clindamycin were started. A thoracentesis was performed on admission with the removal of 1 L of serosanguinous fluid. Pleural fluid showed 57 thousand RBCs/uL and 1750 nucleated cells/uL, comprised of 12% neutrophils and 74% lymphocytes. Pleural fluid glucose was 112 mg/dL, protein 2.9 G/DL, and lactate dehydrogenase (LDH) 92 U/L, but no fluid pH was reported. Pleural adenosine deaminase was 5 U/L, and gram stain did not reveal any pathogens. Serum LDH and protein were 210 U/L and 7.3 g/dL, respectively, consistent with a transudative effusion.

His symptoms improved with diuresis. A PCR of a nasal swab was negative for *Staphylococcus aureus*. On Day 2 of admission, clindamycin and vancomycin were discontinued. Blood cultures from presentation did not show any organisms on gram stain, and no growth was detected. A CXR on Day 4 of admission showed resolution of the right‐sided pleural effusion and no consolidation.

Antinuclear antibody (Ab), double‐stranded deoxyribonucleic acid, rheumatoid factor, myeloperoxidase antibody (MPO), Proteinase 3 Ab (PR3), glomerular basement membrane Ab, Treponema pallidum Ab, 4^th^ generation HIV, hepatitis C Ab, antineutrophil cytoplasmic antibody screen, and cryoglobulins were all negative. Complement component 3 (C3) was 25 mg/dL, and Complement component 4 (C4) was within normal range.

**FIGURE 1:**
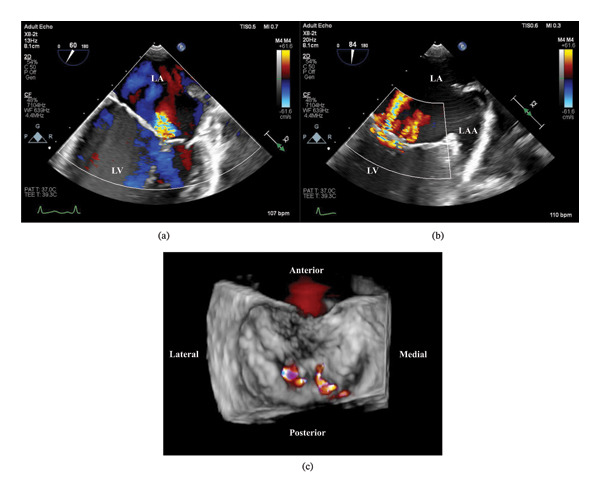
Transesophageal echocardiogram (TEE) images demonstrating diffuse endocardial thickening and valvular thickening, highlighting the mitral valve (MV). (a) Mid‐esophageal (ME) 4‐chamber view of the mitral valve with heterogenous thickening at the tips of the leaflets. Turbulent retrograde flow across the MV visualized. (b) Modified ME 2‐chamber view of the MV and left atrial appendage visualized. Color flow Doppler demonstrating several regurgitant jets across the mitral valve. (c) 3D representation of the mitral valve in a surgical view with applied color flow Doppler. MV thickening is present on the medial portion of the valve, and regurgitant jets are also visualized.

An echocardiogram (transesophageal echocardiography [TTE]) revealed a severely hypokinetic left ventricle (LV) with an ejection fraction (EF) of 35% and severe dilation of the left atrium (LA). Right ventricular wall motion was normal, and the right atrium was dilated. The aortic valve was echodense without stenosis. Mild tricuspid regurgitation was present without stenosis of the tricuspid valve (TV); trace pulmonic regurgitation was present, and there was severe MR without mitral valve (MV) stenosis. A small pericardial effusion was present. TEE showed thickening of the interatrial septum and LA wall, heterogeneous echodense MV leaflet thickening at the tips, moderate MR, thickening of the TV leaflets, and largely unremarkable aortic and pulmonic valves, along with a small pericardial effusion (Figure 1). A CT angiogram of the coronary arteries revealed no evidence of coronary disease. Cardiac magnetic resonance (CMR) imaging demonstrated LV dilation with an EF of 30%, LA dilation, thickening of the MV with associated MR, and no evidence of late gadolinium enhancement (LGE) to suggest prior infarct, inflammation, or infiltrative cardiomyopathy. However, elevated global T2 values in conjunction with elevated native T1 values were suggestive of a diffuse myocardial process with low‐grade acute inflammation indicative of possible myocarditis according to the Lake Louise Criteria [11].

By Day 10 of hospitalization, the patient improved with an unremarkable exam.

Alternative diagnoses of SPAM and ARF with carditis were considered. He was discharged with cefadroxil to complete 14 days of therapy and outpatient follow‐up for monthly penicillin G benzathine 1.2‐million‐unit injections.

On follow‐up, 1 month later, he reported no cardiopulmonary symptoms, and his exam was unremarkable. A repeat TTE demonstrated a dilated LV with diffuse hypokinesis, LVEF of 40%, and mild MR, with overall hemodynamic improvement.

## 3. Discussion

Here, we reported the case of a 21‐year‐old male initially presenting with a GAS bacteremia and positive rapid GAS PCR of the pharynx that developed carditis with moderate to severe MR and heart failure. Alternative diagnoses of SPAM and ARF with carditis were considered.

SPAM lacks validated diagnostic criteria but differs from ARF, in which it is often characterized by acute myocarditis within days of a GAS pharyngitis, typically resolving with antibiotics and minimal sequelae, as opposed to having an onset weeks later [1]. It is thought to result from direct bacterial toxin injury rather than autoimmunity, possibly explaining myocarditis during or shortly after infection [1]. Patients rarely satisfy the revised Jones criteria for ARF [12]. SPAM was first described in the 1940s after autopsies in 12 patients who had GAS pharyngitis, which revealed myocarditis that developed during or within days of the GAS infection, inconsistent with the timeline for ARF [2, 3]. SPAM remains underrecognized due to limited epidemiologic data and a lack of standardized diagnostic criteria, with case reports and series suggesting possible misdiagnosis, underdiagnosis, and inappropriate management as a result [1–4].

A retrospective study of 79 patients with SPAM found that more cases occur among young males with (average age 32 years) few baseline comorbidities, fevers, chills, chest pain, ST segment elevation and/or depression, T wave inversions, elevated cardiac enzymes, and inflammatory markers, with the average onset five days after pharyngitis [1]. Of those that underwent CMR, 97% had subepicardial LGE, and only 1% had documented heart failure. Patients with reduced EF improved during hospitalization, and no recurrences, cardiomyopathy, or death were reported at 1 year follow‐up [1]. None met the revised Jones criteria for ARF.

Other case series reported similar findings, with follow‐up CMR or TTE showing improvement or complete radiographic resolution in subsequent months [2, 3]. While MR has been observed in SPAM, it is not a prominent feature [3, 13]. The literature suggests SPAM should be considered in young males without cardiovascular risk factors who present with chest pain, elevated cardiac biomarkers, ST segment changes, LV wall motion abnormalities, and myocardial inflammation on CMR within 5 days of a GAS pharyngitis, followed by recovery after a short course of antibiotics [1–3, 12, 13]. There is consensus that further study is needed to characterize SPAM’s incidence, pathophysiology, long‐term outcomes, the role of secondary prophylaxis, and its relationship to ARF [1–3, 13]. Notably, SPAM is not included in the 2015 revised Jones criteria or in several major reviews on myocarditis [7, 14–16].

In contrast, ARF follows a GAS pharyngitis and is likely mediated by autoimmunity via molecular mimicry [5, 6]. Symptoms typically develop two to 4 weeks after infection [8]. Carditis, heart failure, shortness of breath, edema, arthritis, arthralgias, Sydenham chorea, subcutaneous nodules, erythema marginatum, and fevers, along with lab and imaging abnormalities, are features of ARF [6, 8, 9]. ARF was the primary cause of death in the early 20^th^ century in the United States (US) among those aged 5−20 years. Current incidence in the US is low (≤ 1/100,000 school‐aged children) but remains much higher in low‐ and middle‐income countries, reaching up to 150 cases per 100,000 individuals [6, 10, 17, 18].

The 2015 Revised Jones Criteria for diagnosing ARF includes five major and four minor criteria [7]. An initial episode of ARF requires two major, or one major plus two minor criteria, along with evidence of a preceding GAS infection. For recurrent ARF, similar criteria apply, but a history of ARF or RHD is required [7]. Arthritis and carditis are the most common major criteria for ARF; however, valvular lesions are the only persistent manifestation and can lead to RHD [5, 9]. Clinical carditis, i.e., murmurs of mitral or aortic regurgitation, occurs in over 50% of ARF cases, with MR and mitral valvulitis being most common [5, 8, 9]. Pancarditis may also be present [7]. Severe MR and heart failure occur in 10%–30% and 10% of initial ARF episodes, respectively [9]. Up to 21% of patients have subclinical carditis, detectable only by echocardiography [7, 9]. Many cases are only later identified as RHD [8]. The 2015 revised Jones criteria now include subclinical carditis as a major criterion [7].

Early antibiotic treatment of GAS pharyngitis can reduce the risk of ARF by up to 80% [9, 10]. Early recognition of ARF with carditis is critical, as secondary prevention with penicillin prophylaxis reduces recurrent ARF and progression to RHD [5, 9]. Whether penicillin prophylaxis improves SPAM outcomes is unclear.

This case highlights diagnostic challenges pertaining to SPAM and ARF, with implications for their management. While the patient was febrile with elevated inflammatory markers during the first admission, direct insight into his cardiac status is lacking in the absence of an EKG, TTE, or cardiac biomarkers. His pulmonary findings may have indicated subclinical carditis and/or heart failure. However, there is incomplete information to evaluate the Jones criteria at that time. During his first admission, he had an active GAS bacteremia and positive GAS PCR of the pharynx, making it difficult to argue that he had ARF. However, the Jones criteria do not define a strict timeline for a “preceding” GAS infection, whereas they define the requisite evidence that one was present prior. This raises the question whether an initial episode of ARF can develop during an active GAS infection, which seems less likely from the pathophysiologic standpoint. Whether recurrent ARF can occur during an active GAS infection is not well addressed, although it seems plausible given that cellular and humoral immunity have already developed. If the patient had undiagnosed RHD or a prior episode of undiagnosed ARF, his presentation could be consistent with recurrent ARF.

However, carditis was confirmed three and a half weeks after his initial admission, within the typical timeframe for ARF and possibly explained by poor medication adherence. Yet, by this time, he was afebrile, and his inflammatory markers did not meet the minor Jones criteria. Considering both admissions together, he met the revised Jones criteria; however, he never strictly met them in either admission alone. The revised Jones criteria do not strictly specify the temporal relationship between the major and minor criteria. Therefore, it is unclear whether he can be diagnosed with ARF. Given his “possible rheumatic fever” as per the revised Jones criteria, secondary prophylaxis was recommended [7]. Asymptomatic pharyngitis does not rule out ARF, considering cases of asymptomatic GAS pharyngitis have been described [5]. Some evidence suggests ARF can develop after GAS skin infections or invasive disease, perhaps relevant in this case [19–21]. The presence of mitral valvulitis with concomitant myocardial inflammation does not negate ARF, as pancarditis can be seen in ARF. Similarly, although heart failure with reduced EF is uncommon in ARF, it is estimated to occur in 10% of cases.

SPAM should be considered as an alternative diagnosis. The patient presentation has many features typical of SPAM. Yet, without clinical data regarding his cardiac status during the first admission, the temporal relationship between his initial GAS infection and the development of carditis is unclear. That there was low suspicion for cardiac involvement at the time may be indicative that SPAM is underappreciated. His CPK was unremarkable during the initial admission, suggesting the absence of myocardial inflammation. However, neither serial cardiac biomarkers nor EKGs were obtained. Insight into his cardiac status is appreciated based on his history of leg swelling, chest pain, and dyspnea that developed after the first admission. However, contrary to the typical course of SPAM, his symptoms and myocarditis developed in three and a half weeks after discharge, despite antibiotics. Although his CMR demonstrated findings consistent with SPAM, he lacked the typical ST segment and cardiac biomarker elevations. He also had MR and heart failure, neither of which has been reported as a typical finding. Confounding the case further was the absence of pharyngitis, inconsistent with the current description of SPAM, but possibly more indicative of our limited understanding of SPAM rather than an argument against it.

In the appropriate context, patients presenting with GAS pharyngitis and myocarditis, SPAM should be considered in the differential diagnosis. Awareness of SPAM is important for multiple reasons, acknowledging that much has yet to be elucidated. First, clinical outcomes seemingly hinge on the use of appropriate antimicrobials. Second, present data suggest sequelae may be limited, an important prognostic consideration and contrast with ARF and carditis. However, data on longitudinal outcomes and optimal treatment duration are lacking. Whether there is a role for secondary prophylaxis in SPAM is also unclear. In this case, monthly injections of penicillin G benzathine were recommended, representing a significant burden to the patient. This highlights the need for further research to optimize treatment. Furthermore, the current description of SPAM is limited to symptomatic GAS pharyngitis; however, the roles of asymptomatic GAS pharyngitis and invasive GAS in SPAM are unclear. Finally, given treatment implications, this case necessitates clarification of the revised Jones criteria definition of a “preceding” GAS infection and whether a recurrent episode of ARF can occur during an active GAS infection.

Whether the patient’s diagnosis is SPAM, ARF with carditis, or an altogether different diagnosis is up for debate. Regardless, given the limited data on SPAM and the rarity of ARF in the US, this case represents a highly unusual one.

## 4. Conclusion

This case highlights the importance of recognizing SPAM as an alternative diagnosis when considering ARF with carditis, with implications for appropriate management. This case also suggests that SPAM may encompass a wider range of clinical presentations than previously described, adding to the call for further research on this entity. Regarding ARF, clarification is needed on the temporal relationship between GAS infection and recurrent ARF, as well as the requisite temporal relationship between the criteria in the revised Jones criteria.

## Author Contributions

All authors were involved in the writing and editing of this case report.

## Funding

This research did not receive any specific grant from funding agencies in the public, commercial, or not‐for‐profit sectors.

## Ethics Statement

Institutional Review Board approval was not required for this study, as it is a single‐patient case report.

## Consent

No identifying information is included in this case report.

## Conflicts of Interest

The authors declare no conflicts of interest.

## Data Availability

Data sharing is not applicable to this article as no datasets were generated or analyzed during the current study.
